# Predictive value of CHADS_2_ and CHA_2_DS_2_-VASc scores for acute myocardial infarction in patients with atrial fibrillation

**DOI:** 10.1038/s41598-017-04604-w

**Published:** 2017-07-05

**Authors:** Hui Pang, Bing Han, Qiang Fu, Zhenkun Zong

**Affiliations:** 1 Department of Cardiology, Xuzhou Central Hospital, Xuzhou Clinical School of Xuzhou Medical College, Affiliated Xuzhou Hospital of Medical College of Southeast University, Xuzhou, Jiangsu China; 2 Department of Neurosurgery, Affiliated Hospital of Xuzhou Medical College, Xuzhou, Jiangsu China

## Abstract

The presence of acute myocardial infarction (AMI) confers a poor prognosis in atrial fibrillation (AF), associated with increased mortality dramatically. This study aimed to evaluate the predictive value of CHADS_2_ and CHA_2_DS_2_-VASc scores for AMI in patients with AF. This retrospective study enrolled 5140 consecutive nonvalvular AF patients, 300 patients with AMI and 4840 patients without AMI. We identified the optimal cut-off values of the CHADS_2_ and CHA_2_DS_2_-VASc scores each based on receiver operating characteristic curves to predict the risk of AMI. Both CHADS_2_ score and CHA_2_DS_2_-VASc score were associated with an increased odds ratio of the prevalence of AMI in patients with AF, after adjustment for hyperlipidaemia, hyperuricemia, hyperthyroidism, hypothyroidism and obstructive sleep apnea. The present results showed that the area under the curve (AUC) for CHADS_2_ score was 0.787 with a similar accuracy of the CHA_2_DS_2_-VASc score (AUC 0.750) in predicting “high-risk” AF patients who developed AMI. However, the predictive accuracy of the two clinical-based risk scores was fair. The CHA_2_DS_2_-VASc score has fair predictive value for identifying high-risk patients with AF and is not significantly superior to CHADS_2_ in predicting patients who develop AMI.

## Introduction

Atrial fibrillation (AF) is a common cardiac rhythm disturbance with an age-related increase in both women and men. The arrhythmia is also a major cardiac cause of stroke^1^. Given that AF has become a major cardiovascular challenge in the last two decades, it is crucial to have an updated picture of its medical, social and economic impact of AF to plan appropriate interventions^2^.

The CHADS_2_ and CHA_2_DS_2_-VASc scoring systems have been proved efficacy to stratify stroke and thromboembolism risk in patients with non-valvular AF (NVAF). According to recent large clinical trials, patients identified as high-risk category using the CHADS_2_ score comprise many AF patients at risk of fatal and devastating strokes. Thus, the CHA_2_DS_2_-VASc score is recommended by current major guidelines to identify the “truly low-risk patients” with AF^3^. Except for preventing stroke in AF patients, the CHADS_2_ and CHA_2_DS_2_-VASC scores have been reported recently to predict cardiovascular^4^ and cerebrovascular events^5^.

Coronary artery disease (CAD) and AF have close relationship and interact with each other. CAD is considered a risk factor for AF as well as a disease for which the adverse outcome is modulated by AF. Reports from several clinical trials have proved that AF is associated with an increased risk of incident MI^6–8^. Furthermore, the coexistence of the two diseases increases the risk of future cardiovascular events and stroke dramatically^9^. Subsequently, the number and complexity of therapies increase and the potential for significant adverse interactions grow. In addition, CAD and stroke share a number of common cardiovascular risk factors, including sex, age, obesity, hypertension, diabetes mellitus (DM), and congestive heart failure (CHF). Identification of high-risk AF patients is a needed first step to develop cost-effective approaches for prevention of CAD. For example, Ankle-Brachial Index (ABI) is a non-invasive tool in evaluating cardiovascular risk, useful in predicting MI in NVAF patients^10^. The CHADS_2_ and CHA_2_DS_2_-VASc scores include similar risk factors for the development of CAD. Therefore, unsurprisingly, there have been recent reports about the two stroke risk scoring systems used to predict severity or even outcome of CAD^11^. Most previous studies mainly focused on AF after CAD and demonstrated AF adversely influences the outcomes in patients with CAD. However, studies concerned with the cardiovascular risk stratification and identification AF patients at higher risk to experience acute myocardial infarction (AMI) using the CHADS_2_ and CHA_2_DS_2_-VASc scores are relatively sparse. The objective of our study was to investigate the predictive value of CHADS_2_ and CHA_2_DS_2_-VASc scores for AMI risk in AF, and subsequently compare the accuracy of the CHADS_2_ score with CHA_2_DS_2_-VASc score in predicting the AMI incidence.

## Subjects and Methods

This was a retrospective study based on electronic hospital databases of our hospital. This study enrolled 5140 consecutive patients with NVAF who presented to our department of cardiology from November 2013 to October 2016. The definition of NVAF was in accordance with 2014 AHA/ACC/HRS guideline for the management of patients with AF^1^. Patients were excluded if they had rheumatic mitral stenosis, a mechanical or bioprosthetic heart valve, or mitral valve repair, and death in hospital. To be eligible for our study, AMI was comprised of ST-segment elevation myocardial infarction (STEMI) and non-STEMI within the previous 30 days. The following information was collected from the database: age, gender, history of CAD, CHF, hypertension, DM, stroke or transient ischemic attack (TIA), thromboembolism, vascular disease (including prior myocardial infarction, peripheral artery disease, or complex aortic plaque), hyperlipidaemia, hyperuricemia, hyperthyroidism, hypothyroidism and obstructive sleep apnea (OSA). From the baseline clinical characteristics of each patient, the pre-AMI CHADS_2_ and CHA_2_DS_2_-VASc scores were calculated according to the ESC guidelines for the management of AF^12^. The study was approved by the research ethics committee of Xuzhou Central Hospital and all patients provided their written informed consent. All methods were carried out in accordance with the approved guidelines and regulations.

### Statistical Analysis

Quantitative variables are expressed as mean ± standard deviation for parametric variables and were analyzed using the independent-samples t test. In addition, nonparametric variables are presented as median with interquartile ranges. Categorical variables are expressed as numbers and percentages, and were compared using the chi-square test. The relationships between CHADS_2_ score and AMI rate as well as the relationships between CHA_2_DS_2_-VASc score and AMI rate were examined by Kruskal-Wallis non-parametric H test. The multivariate logistic regression analysis was used to evaluate the associations of the baseline clinical characteristics, CHADS_2_ and CHA_2_DS_2_-VASc scores with the prevalence of AMI, respectively. We identified the optimal cut-off values of the CHADS_2_ and CHA_2_DS_2_-VASc scores each based on receiver operating characteristic (ROC) curves to predict the risk of AMI. The area under the curve (AUC) is a rough guide for quantifying the discriminatory capacity of a diagnostic test ranked as: excellent (0.9–1), good (0.8–0.89), fair (0.7–0.79), poor (0.6–0.69), or fail/no discriminatory capacity (0.5–0.59)^13^. The differences between the areas under the two ROC curves were assessed by a univariate z-score test. The agreement on identification of AMI risk determined by the two scores was tested by the Cohen’s kappa coefficient (κ) and McNemar test was used to compare the differences in risk. The agreement of κ 0.81–1.00 is interpreted as almost perfect agreement, 0.61–0.80 as substantial agreement, 0.41–0.60 as moderate agreement, 0.21–0.40 as fair agreement, and ≤0.20 as slight agreement^14^. Assessing value of CHADS_2_ and CHA_2_DS_2_-VASc scores in AMI prediction using net reclassification improvement (NRI) and integrated discrimination improvement (IDI)^15^. Whether NRI and IDI were statistically significant was analyzed using the Z-test. The analyses were performed using the SPSS 21.0 and ROCKIT 0.9β statistical software, and a two-sided *P*-value < 0.05 was considered statistically significant.

## Results

A total of 5140 AF patients were enrolled in this study. These patients were divided into two groups: 300 in the AMI group and 4840 in the non-AMI group. Patient demographics, cardiovascular diseases and cardiovascular risk factors of the two groups are shown in Table 1. Of the 300 AMI patients, 87 (29.0%) were female, with a mean age of 67.4 ± 10.9 years. But in the non-AMI group, 1867 (38.6%) were female, with a mean age of 59.9 ± 12.4 years. In comparison with the non-AMI group, the AMI group had a higher prevalence of hypertension, DM, CHF, CAD, prior MI, stroke/TIA, thromboembolism, vascular disease and hyperlipidaemia. AMI rate positively correlated with the CHADS_2_ score and the CHA_2_DS_2_-VASc score.

**Table 1:** Characteristics of the study population.

Characteristics	Total sample (n = 5140)	AMI (n = 300)	Non-AMI (n = 4840)	*P-*value
Age, years	60.3 ± 12.4	67.4 ± 10.9	59.9 ± 12.4	<0.001
Components of the CHADS_2_ score				
Age				
<54 years	1414 (27.5)	34 (11.3)	1380 (28.5)	<0.001
54–63 years	1512 (29.4)	65 (21.7)	1447 (29.9)	0.002
64–74 years	1615 (31.4)	117 (39.0)	1498 (31.0)	0.004
≥75 years	599 (11.7)	84 (28.0)	515 (10.6)	<0.001
Female	1954 (38.0)	87 (29.0)	1866 (38.6)	0.001
Hypertension	2116 (41.2)	191 (63.7)	1925 (39.8)	<0.001
Diabetes mellitus	797 (38.0)	97 (32.3)	700 (14.5)	<0.001
Congestive heart failure	2226 (43.3)	277 (92.3)	1949 (40.3)	<0.001
NYHA class				
I	241 (4.7)	128 (42.7)	113 (2.3)	<0.001
II	773 (15.0)	68 (22.7)	705 (14.6)	<0.001
III	681 (13.2)	45 (15.0)	636 (13.1)	0.357
IV	526 (10.2)	36 (12.0)	490 (10.1)	0.298
Coronary artery disease	1070 (20.8)	106 (35.3)	964 (19.9)	<0.001
Prior myocardial infarction	396 (7.7)	51 (17.0)	345 (7.1)	<0.001
Stroke/Transient ischemic attack	417 (8.1)	54 (18.0)	363 (7.5)	<0.001
Thromboembolism	175 (3.4)	3 (1.0)	172 (3.6)	0.018
Vascular disease	128 (2.5)	14 (4.7)	114 (2.4)	0.013
Comorbidities				
Hyperlipidaemia	1061 (20.6)	111 (37.0)	950 (19.6)	<0.001
Hyperuricemia	45 (0.9)	2 (0.7)	43 (0.9)	0.689
Hyperthyroidism	80 (1.6)	5 (1.7)	75 (1.5)	0.874
Hypothyroidism	56 (1.1)	4 (1.3)	52 (1.1)	0.675
Obstructive sleep apnea	58 (1.1)	2 (0.7)	56 (1.2)	0.435
CHADS_2_ score	1.0 (1.0–2.0)	2.0 (2.0–3.0)	1.0 (0.0–2.0)	<0.001
0	1231 (23.9)	3 (1.0)	1228 (25.4)	<0.001
1	2285 (44.5)	71 (23.7)	2214 (45.7)	<0.001
2	944 (18.4)	89 (29.7)	855 (17.7)	<0.001
3	416 (8.1)	71 (23.7)	345 (7.1)	<0.001
4	179 (3.5)	39 (13.0)	140 (2.9)	<0.001
5	75 (1.5)	23 (7.7)	52 (1.1)	<0.001
6	10 (0.2)	4 (1.3)	6 (0.1)	<0.001
CHA_2_DS_2_-VASc score	2.0 (1.0–3.0)	4.0 (2.3–5.0)	2.0 (1.0–3.0)	<0.001
0	645 (12.5)	1 (0.3)	644 (13.3)	<0.001
1	1235 (24.0)	31 (10.3)	1204 (24.9)	<0.001
2	1297 (25.2)	43 (14.3)	1254 (25.9)	<0.001
3	907 (17.6)	71 (23.7)	836 (17.3)	<0.001
4	573 (11.1)	53 (17.7)	520 (10.7)	<0.001
5	281 (5.5)	53 (17.7)	228 (4.7)	<0.001
6	137 (2.7)	34 (11.3)	103 (2.1)	<0.001
7	50 (1.0)	10 (3.3)	40 (0.8)	<0.001
8	15 (0.3)	4 (1.3)	11 (0.2)	0.001

Data given as mean ± SD, n (%) or median (IQR). Abbreviations: AMI, acute myocardial infarction. NYHA, New York Heart Association.

Multivariable models of the difference between baseline clinical characteristics and prevalence of AMI in patients with AF are shown in Table 2. Multivariate logistic regression analysis showed that advanced age, male, and history of hypertension, DM, hyperlipidaemia, stroke/TIA and prior MI were independent risk factors for AMI (P < 0.05). The associations of CHADS_2_ and CHA_2_DS_2_-VASc scores with the prevalence of AMI were assessed by multivariable logistic regression in different models (Table 3). The unadjusted logistic regression analysis revealed that both CHADS_2_ score (odds ratio 2.166, 95%CI 1.987–2.362, P < 0.001) and CHA_2_DS_2_-VASc score (odds ratio 1.673, 95%CI 1.566–1.789, P < 0.001) were associated with an increased odds ratio of the prevalence of AMI in patients with AF. Furthermore, the adjusted logistic regression analysis revealed that both CHADS_2_ score (odds ratio 2.120, 95%CI 1.942–2.315, P < 0.001) and CHA_2_DS_2_-VASc score (odds ratio 1.639, 95%CI 1.532–1.753, P < 0.001) were associated with an increased odds ratio of the prevalence of AMI in patients with AF, after adjustment for hyperlipidaemia, hyperuricemia, hyperthyroidism, hypothyroidism and OSA.

**Table 2:** Associations between baseline clinical characteristics and prevalence of acute myocardial infarction in patients with atrial fibrillation.

Variable	Multivariate analysis	
	OR (95%CI)	*P-*value
Age	1.047 (1.035–1.060)	<0.001
Male	1.648 (1.263–2.150)	<0.001
Hypertension	1.521 (1.171–1.976)	0.002
Diabetes mellitus	1.884 (1.443–2.462)	<0.001
Hyperlipidaemia	1.764 (1.362–2.285)	<0.001
Stroke/Transient ischemic attack	1.770 (1.274–2.458)	0.001
Prior myocardial infarction	1.409 (1.004–1.977)	0.047

OR, odds ratio; CI, confidence interval.

**Table 3:** Associations of CHADS_2_ and CHA_2_DS_2_-VASc scores with prevalence of acute myocardial infarction in patients with atrial fibrillation.

Score	Unadjusted OR (95% CI)	*P-*value	Adjusted OR (95% CI)	*P-*value
CHADS_2_				
Per 1-point increase	2.166 (1.987–2.362)	<0.001	2.120 (1.942–2.315)	<0.001
CHA_2_DS_2_-VASc				
Per 1-point increase	1.673 (1.566–1.789)	<0.001	1.639 (1.532–1.753)	<0.001

OR, odds ratio; CI, confidence interval. Adjusted analyses were controlled for hyperlipidaemia, hyperuricemia, hyperthyroidism, hypothyroidism and obstructive sleep apnea.

**Figure 1:**
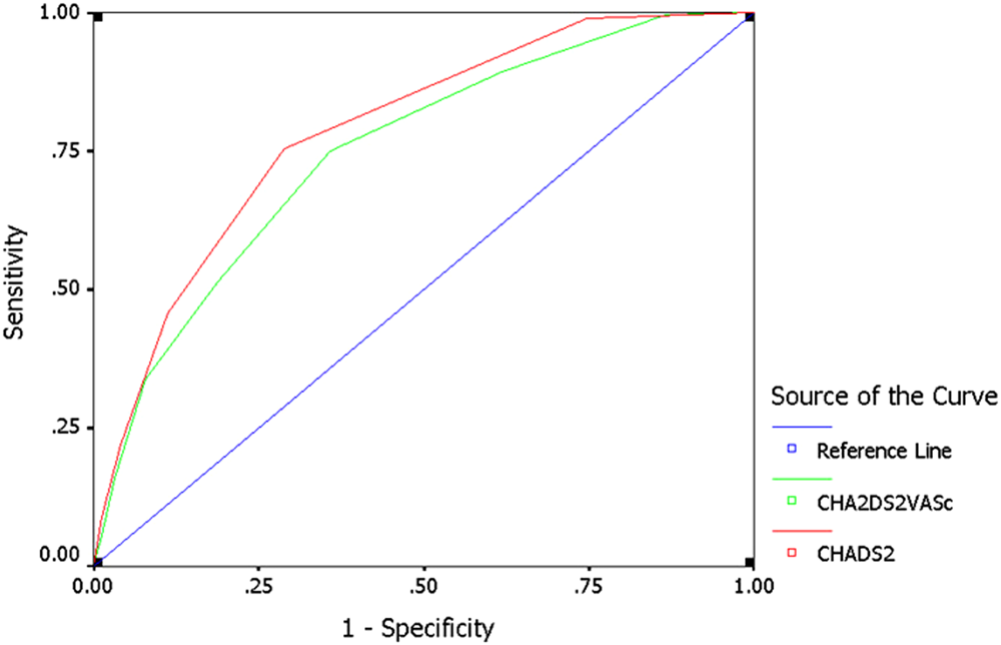
Receiver operating characteristic curves (ROC) for CHADS_2_ and CHA_2_DS_2_-VASc scores for prediction of acute myocardial infarction (AMI) in patients with atrial fibrillation (AF). The area under the receiver operating characteristic curve (AUC) for CHADS_2_ score predicting AMI is 0.787 (0.763–0.812), *P* < 0.001. The sensitivity and specificity for a CHADS_2_ score ≥2 are 75.3% and 71.1%, respectively. The AUC for CHA_2_DS_2_-VASc score is 0.750 (0.722–0.777), *P* < 0.001. The sensitivity and specificity for a CHA_2_DS_2_-VASc score ≥3 are 75.0% and 64.1%, respectively. The difference between the two areas under the curves is not significant (*P* > 0.05).

ROC analysis indicated that the AUC for CHADS_2_ score predicting AMI was 0.787 (0.763–0.812), *P* < 0.001 (Fig. 1). The optimal cutoff value was CHADS_2_ ≥ 2, with a sensitivity of 75.3% and a specificity of 71.1%. The AUC for CHA_2_DS_2_-VASc score predicting AMI was 0.750 (0.722–0.777), *P* < 0.001. The optimal cutoff value for CHA_2_DS_2_-VASc score displaying the best predictive value was ≥3, with a sensitivity of 75.0% and a specificity of 64.1%. The difference between the two areas under the curves was not significant (Z = 1.947, *P* > 0.05). To follow up on this reclassification, we calculated category-based NRI and absolute IDI. The CHA_2_DS_2_-VASc score was chosen as reference. The CHADS_2_ score resulted in a NRI of 0.069 (*P* = 0.002) and an IDI of 0.074 (*P* > 0.05).

The risk estimates of AMI were stratified by the cut-offs of CHADS_2_ and CHA_2_DS_2_-VASc scores, using the baseline characteristics of 5140 AF patients, respectively. CHADS_2_ score rated 1624 patients (31.6%) as having high risk of AMI, versus 1941 (37.8%) who were considered high risk by CHA_2_DS_2_-VASc score. Both CHADS_2_ and CHA_2_DS_2_-VASc scores indicated that 1418 (27.6%) patients were at high risk of AMI and 2993 (58.2%) patients were at low risk of AMI. CHA_2_DS_2_-VASc score rated 523 patients (10.2%) as having high risk of AMI, who were considered low risk by the CHADS_2_ score. On the contrary, CHADS_2_ score rated 206 (4.0%) patients at high risk of AMI, but considered low risk by the CHA_2_DS_2_-VASc score. The Kappa coefficient showed substantial concordance between the two tests (Kappa = 0.688, *P* < 0.001) and the consistency rate between the two scores was 85.8%. The differences in risk discrimination of high risk AMI patients between the two scores were significant (McNemar test, *P* < 0.001). The AMI rate was significantly higher in patients with CHADS_2_ ≥ 2 compared with those with CHADS_2_ < 2 (13.9% [226/1624] versus 2.1% [74/3516], P < 0.001). In addition, patients with CHA_2_DS_2_-VASc ≥ 3 had a significantly higher AMI compared with patients with CHA_2_DS_2_-VASc < 3 (11.6% [225/1941] versus 2.3% [75/3199], P < 0.001).

## Discussion

The major findings of this study are as follows: (i) AMI rate positively correlates with the CHADS_2_ and CHA_2_DS_2_-VASc scores; (ii) CHADS_2_ and CHA_2_DS_2_-VASc scores are independent predictors of AMI in AF patients; (iii) accuracy of CHADS_2_ score in predicting high-risk AF patients who developed AMI is similar to CHA_2_DS_2_-VASc score; (iv) the predictive accuracy of the two scores is fair; (v) the best cutoff value to predict AMI is CHADS_2_ ≥ 2 or CHA_2_DS_2_-VASc ≥ 3. The study suggests that CHADS_2_ and CHA_2_DS_2_-VASc scores may be useful as AMI risk indices for patients with AF, although the statistical impact is fair.

AF and CAD are closely associated. Data from recent studies in different patient populations have shown that AF and CAD coexist in a large percentage of patients (18–34%)^16^. CAD can promote AF due to its setting of consequential physiopathological changes, including inflammation, fibrosis, hypertrophy and atrial ischemia^1^. AF population with a history of CAD has been reported to be associated with recurrent AF episodes, heart failure and increased short-term and long-term mortality^17^. Furthermore, the morbidity and mortality of MI-associated stroke is often high, and the risk of post-MI stroke may be highest over the first 3 months^18^. Stable CAD was common in Chinese AF patients who were more likely to be older and to have more co-morbidities. Additionally, stable CAD was strongly associated with a higher risk of 1-year all-cause mortality^19^. A previous clinical trial proved that coronary artery calcium was independently associated with an increased risk of AF^20^. But whether CAD is the underlying pathophysiologic link between coronary artery calcium and AF is still controversial^21^.

AF patients with CAD have more risk factors and comorbidities than patients without CAD. Increased risk of stroke and other thrombo-embolic events, left ventricular dysfunction, aggravation of heart failure and hospitalizations related to AF complicating MI result in significant reduced exercise capacity, degraded quality of life and long-term death. For instance, the development of AF during index hospitalisation for MI was associated with increased risk of sudden cardiac death^22^. Coexistence of atherosclerotic risk factors, systemic inflammation and platelet activation can promote a pro-thrombotic state and eventually MI in AF. In AF patients, impaired artery dilatation predisposes to atherosclerotic complications is associated with increased risk of cardiovascular events^23^. There are other mechanisms for the increased MI risk in patients with AF. For example, episodes of poorly controlled AF with high ventricular rates may result in type 2 MI^24^. Shibata *et al*. reported that AF was the most frequent cause of coronary artery embolism which was recognized as an important nonatherosclerotic cause of AMI^25^.

The data from our study demonstrates that, the prevalence of hypertension, DM, CHF, CAD, prior MI, stroke/TIA, thromboembolism, vascular disease and hyperlipidaemia among 300 AF patients with AMI were higher than the patients without AMI. The present data also indicated that advanced age, male, and history of hypertension, DM, hyperlipidaemia, stroke/TIA and prior MI were independent risk factors for AMI in AF patients.

Much earlier prevention of the MI might reduce the burden of AF to protect the patients, not only from the progression of AF from an easily treated condition to an utterly refractory problem, but also from the risk of bleeding with triple therapy (vitamin K antagonist, aspirin, and clopidogrel). It is an enormous challenge to the management of AF patients at risk or with previous MI, because of the complexity of antithrombotic treatment to prevent both thromboembolic and cardiac events. Previous studies showed that oral anticoagulants alone were not enough to lower the risk of MI in AF^26^. Of note, recent studies have demonstrated that combined anticoagulant and antiplatelet therapy is independently associated with significantly increased risk for bleeding compared with anticoagulant therapy alone in AF patients^27^. Moreover, the antithrombotic treatment of AF may complicate coronary revascularization and related antiplatelet treatment^11^. Therefore, the attention must be directed towards preventing CAD at an early stage in order to improve the treatment and prognosis of AF, with more focus on the AMI risk stratification. Our data illustrated that AMI rate positively correlated with the CHADS_2_ score and the CHA_2_DS_2_-VASc score. Furthermore, both CHADS_2_ score and CHA_2_DS_2_-VASc score were independently associated with an increased prevalence of AMI.

Except for preventing stroke in AF patients, several studies recently have reported that the CHADS_2_ and CHA_2_DS_2_-VASc scores can also predict severity and outcomes of stroke and thromboembolic events^28^ in patients with AF and those without AF^29, 30^. Compared with HF with concomitant AF, the risk of thromboembolism was higher among HF patients without AF at high CHA_2_DS_2_-VASc scores^31^. In eight cohort studies (7 prospective and 1 retrospective) of 31,509 patients with CAD, CHADS_2_ score was associated with increased mortality and stroke/TIA incidence in patients without AF. But no significant association was found between CHADS_2_ score and stroke/TIA incidence in patients with AF^32^. Furthermore, CHA_2_DS_2_-VASc score was strongly predictive of stroke and embolic events in a retrospective cohort of 465 patients with cardiac myxomas following surgical treatment^33^. Hoshino T *et al*. found that CHADS_2_ and CHA_2_DS_2_-VASc scores were useful in predicting functional status after stroke in CAD patients^34^. Both the CHADS_2_ and CHA_2_DS_2_-VASc scores were used to predict contrast-induced nephropathy (CIN) in patients with CAD who underwent urgent percutaneous coronary intervention (PCI)^35^. Chou RH *et al*. enrolled 539 stable CAD patients who underwent elective PCI and reported that CHADS_2_ score independently increased the risk of CIN. Moreover, the predictive accuracy of CHADS_2_ score was not inferior to either R_2_CHADS_2_ score or Mehran’s risk score^36^. In addition, Subjects who underwent coronary artery bypass surgery with higher CHADS_2_ scores had significantly higher all-cause mortality and cardiovascular mortality^37^. The present results showed that the AUC for CHADS_2_ score was 0.787 with a similar accuracy of the CHA_2_DS_2_-VASc score (AUC 0.750) in predicting “high-risk” AF patients who developed AMI. Adding extra points for MI (e.g. age ≥75, prior myocardial infarction, etc.) did not improve the predictive accuracy of CHA_2_DS_2_-VASc. However, the predictive accuracy of the two clinical-based risk scores was fair (AUC 0.7–0.79). Although the CHA_2_DS_2_-VASc score might be more inclined to classify AF patients as high-risk AMI than the CHADS_2_ score did. The discriminative and reclassification power of CHADS_2_ and CHA_2_DS_2_-VASc scores was assessed using NRI and IDI. When CHADS_2_ score ≥2 and CHA_2_DS_2_-VASc score ≥3 were chosen as the best predictive cutoff values, CHADS_2_ score significantly improved risk classification for AMI by assessment of NRI. However, insignificant IDI for CHADS_2_ score was showed. Now that the CHADS_2_ and CHA_2_DS_2_-VASc scores have proved useful not only to assess the stroke, but also to assess the AMI risk, and to drive therapeutic choices, our future clinical trials are going to examine the usefulness of addition of antiplatelet drugs to oral anticoagulants in reducing the risk of both MI and stroke in AF patients.

### Study limitations

The main limitation of this study is related to its retrospective nature. The history of AMI was ascertained at the time of admission. CHADS_2_ and CHA_2_DS_2_-VASc scores were calculated retrospectively after the AMI had occurred. Precise information about the pre-AMI status was unavailable. It should also be noted that, because this was a hospital-based observational study, the characteristics of the patients with AF admitted to the Department of cardiology, Xuzhou Central Hospital might differ from those of the general population. Additionally, these data derived from a single medical center survey might exist selection bias. Although we adjusted for several variables, residual and unmeasured confounding might not be fully reflected. Treatment related to the incidence of AMI was not studied, especially for anticoagulant and antiplatelet therapy. Thus, we could not exclude the possibility that treatment influenced predictive value of the two scores. Finally, since all patients in our study were Asians, the results were not directly translatable to other ethnicities. As a result, further prospective multicenter and larger-scale studies are needed to clarify our conclusions.

## Conclusions

Focusing specifically on risk stratification of AMI by the CHADS_2_ and CHA_2_DS_2_-VASc scores as well as means for optimizing outcomes in the treatment of AF is the significance of our study. Even if the accumulated evidence has shown that CHA_2_DS_2_-VASc is better at identifying ‘truly low-risk’ patients with AF who develop stroke and thromboembolism, our data demonstrate that CHA_2_DS_2_-VASc is not significantly superior to CHADS_2_ for predicting AMI in AF patients.
